# Influenza in long-term Dutch travelers in the tropics: symptoms and infections

**DOI:** 10.1186/s12879-016-1502-6

**Published:** 2016-04-16

**Authors:** Jane Whelan, Guus F. Rimmelzwaan, Anneke van den Hoek, Sanne-Meike Belderok, Gerard J. B. Sonder

**Affiliations:** Department of Infectious Diseases, Public Health Service Amsterdam, Nieuwe Achtergracht 100, 1018WT Amsterdam, The Netherlands; Academic Medical Centre, Amsterdam, The Netherlands; Erasmus Medical Center, Rotterdam, The Netherlands; National Coordination Centre for Traveler’s Health Advice, Amsterdam, The Netherlands

**Keywords:** Influenza, Seroconversion, Attack rate, Travelers

## Abstract

**Background:**

Influenza is a common infection among travelers, and attack rates are well documented in short-term travelers and holiday makers. Little data exists on long-term, non-expatriate travelers.

**Methods:**

This was a prospective mono-centre study of immunocompetent, Dutch travelers aged ≥18 to 64 years. It was conducted at the Public Health Service travel clinic in Amsterdam from December 2008 to September 2011, and included all travelers intending to travel to a tropical or sub-tropical country.

**Results:**

Among 602 Dutch long-term travelers to tropical regions, 82 % had protective influenza antibody titres pre-travel. The influenza attack rate of serologically confirmed infection during travel was 15 %, and of symptomatic infection was 6.3 % (fever alone) and 2 % (ILI), respectively.

**Conclusions:**

The attack rate in this study is similar to seasonal rates of infection in the general population. Influenza vaccination pre-travel is therefore most important for people at risk of medical complications due to influenza.

## Background

Influenza is the most frequently reported vaccine-preventable disease affecting visitors to (sub) tropical countries [1]. In 2009, an increase in the proportion of travel-related respiratory disease in Europe, mostly attributable to pandemic A(H1N1) influenza, was recorded [2]. The impact of influenza virus infection on travelers in terms of morbidity, healthcare accessed and hospitalization, is well documented [3] in short-term holiday makers, business travelers, and other traveler groups (Hadj pilgrims [4], cruise-ship passengers [5]). Travelers are also an acknowledged risk for onward transmission while in transit and at home [6]. Western adults are traveling in unprecedented numbers to emerging economies (often tropical and subtropical regions), for prolonged periods for ‘gap years’, to work, study, and tour. In this study of Dutch long-term travelers (3 to 12 months) to (sub) tropical regions, we prospectively document the attack rate (AR) of influenza infection and the incidence of influenza like illness (ILI) while traveling, and identify risk factors associated with symptomatic and asymptomatic infection from 2009 to 2011.

## Methods

**Fig. 1:**
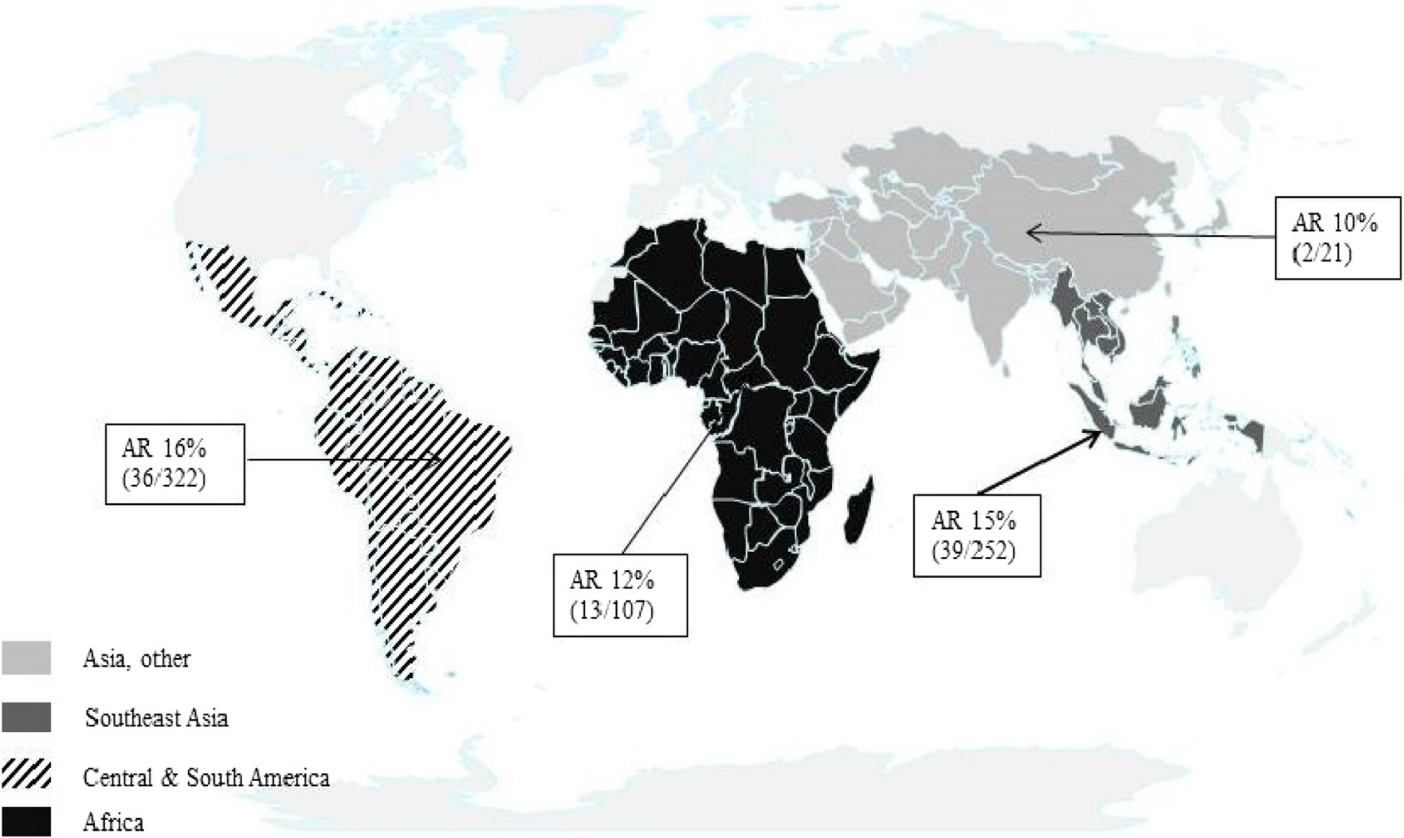
Influenza transmission regions and influenza attack rates. Countries where travelers spent the majority of their trip were categorized into Influenza Transmission Zones and then, for analysis, into 4 regions: South East Asia, Africa, Central and Latin America and “Asia (Other)”

A prospective mono-centre study of immunocompetent, Dutch travelers aged ≥18 to 64 years was conducted at the Public Health Service travel clinic in Amsterdam from December 2008 to September 2011. All clients planning to travel to any (sub) tropical country in Africa, Central America, the Caribbean, South America or Asia for ≥12 and ≤52 weeks were invited to participate. Pre-travel, clients were interviewed by a nurse or physician and asked about travel purpose (work/study or tourism), travel duration, planned destination(s), and demographic details. No influenza vaccination history was established, but in the Netherlands, influenza vaccination is not recommended for travelers or healthy, young adults. At the time of the study, vaccination was only recommended for people ≥ 65 years and people with certain chronic diseases. Participants were given a digital thermometer (Huikeshoven Medical, Tiel, The Netherlands) and asked to take their temperature if they felt feverish while traveling. They kept a structured, weekly travel diary, recording their itinerary, symptoms experienced including respiratory, gastrointestinal and other systemic complaints and physician visits while ill. Diaries were completed on paper or digitally. Travelers received a weekly email reminder and were seen 2 to 6 weeks after return. Blood samples were taken before and after travel and paired samples were tested simultaneously post-travel for antibodies against influenza viruses using the hemagglutination-inhibition (HI) assay [7, 8]. Vaccine strains used represented the epidemic strains circulating worldwide during the study period: A(H1N1pdm09), A(H3N2brisbane), A(H3N2perth), A(H1N1brisbane), B/Florida and B/Brisbane [9]. Influenza infection was defined as a ≥4-fold rise in antibody titre post-travel from a pre-travel titre of ≥10, or if pre-travel titer was <10 titer, a post-travel titer of ≥40. Fever was defined as thermometer-confirmed body temperature >38.0 °C and influenza like illness (ILI) was defined as fever and cough or sore throat in the absence of other diagnoses. Primary regions visited while travelling were categorized into Influenza Transmission Zones [10] and then into 4 regions: South East Asia, Africa, Central and Latin America and “Asia (Other)” (Fig. 1).

Differences in the proportion of confirmed influenza infections by sex, age-group, primary influenza transmission zone visited, fever >38.0 °C and ILI were tested using Pearson’s chi-squared for each influenza strain. Positive predictive value (PPV) for seroconversion was the proportion of symptomatic cases seroconverting for infection. The association between infection and ILI was tested using logistic regression and odds ratios with 95 % confidence intervals. Incidence of fever and ILI while travelling was estimated as 1st episodes of fever or ILI per 100 person-weeks of travel with Poisson distributed 95 % confidence intervals (STATA version 13).

## Results

### Study population

Overall, 602 respondents were included. The median age was 25 years (IQR:23–30), and 64 % (*n* = 389) were female; median travel duration was 20 weeks (IQR:16–25 weeks). Travelers’ primary destinations (influenza transmission zone) are listed in Table 1. Travel periods were: 228 (38 %) travelled in 2009 (earliest departure: 9 December 2008), 309 (51 %) through 2010, 255 (42 %) through 2011 and 47 (8 %) through 2012 (latest return 01 September 2012). Pre-travel, 82 % (*n* = 496) had protective influenza antibody titres: 214 (36 %) for A(H1N1pdm09), 264 (44 %) for A(H3N2brisbane), 283 (47 %) for A(H3N2perth), 202 (34 %) for A(H1N1brisbane), 208 (35 %) for B/Florida and 68 (11 %) for B/Brisbane. Of those who departed before the ‘public health emergency of international concern’ (pandemic) was formally declared on 25 April 2009 [11] (*n* = 107), 22 % were already positive for A(H1N1pdm09) as early as January 2009. There was no difference in age or gender compared to those who were H1N1pdm09 negative pre-travel.

**Table 1:** Cross-tabulations of seroconversion for influenza viruses circulating worldwide during the study period for *n* = 602 long-term Dutch travelers, 2009 to 2012

Variables	Total respondents N	Seroconversion for any virus^a^			A/California/007/09 H1N1pdm [H1pdm09]			A/Brisbane/59/07 [H1N1]			A/Brisbane/10/07 [H3N2]			A/Perth/16/09 [H3N2]			B/Florida/4/06			B/Brisbane/60/08		
		*n*	n/N%	*p* value	*n*	n/N%	*p* value	*n*	n/N%	*p* value	*n*	n/N%	*p* value	*n*	n/N%	*p* value	*n*	n/N%	*p* value	*n*	n/N%	*p* value
Total	602	90	15	N/A	43	7	N/A	16	3	N/A	26	4	N/A	34	6	N/A	12	2	N/A	11	2	N/A
Age-group																						
<25 years of age	249	36	14		16	6		5	2		9	4		17	7		4	2		3	1	
25–28 years	169	39	23		22	13		7	4		10	6		9	5		5	3		5	3	
> = 29 years	184	15	8	<0.001^*^	5	3	0.001^*^	4	2	0.366	7	4	0.482	8	4	0.531	3	2	0.571	3	2	0.410
Gender																						
Female	389	63	16		31	8		12	3		17	4		24	6		5	1		5	1	
Male	213	27	13	0.247	12	6	0.287	4	2	0.379	9	4	0.933	10	5	0.454	7	3	0.093	6	3	0.180
Major travel flu zone																						
Southeast Asia	252	39	15		16	6		3	1		11	4		17	7		5	2		5	2	
Central & South America	222	36	16		20	9		10	5		9	4		10	5		5	2		5	2	
Asia, other	21	2	10		1	5		0	0		0	0		0	0		1	5		0	0	
Africa	107	13	12	0.687	6	6	0.580	3	3	0.132	6	6	0.702	7	7	0.469	1	1	0.679	1	1	0.771

Age was summarized into tertiles: <25 years, 25–28 years and > =29 years. N/A Not Applicable

^a^In 10 individuals where the pre-travel titre was ≥40 there was evidence of re-infection (4 fold rise in antibody titre)

*P* value throughout is Pearson’s chi-squared; Significance assigned (*) at *p* < 0.01

### Influenza symptoms and infections

Of all 602 travelers, 209 (35 %) had fever while traveling with a median temperature of 38.5 °C (range 38.0 to 41.4 °C). Five percent (*n* = 32/602) met the definition for ILI of whom seven complained of 2 ILI episodes. A further *n* = 149 with fever also complained of headache, retrosternal pain or myalgia but did not meet the ILI definition. Of ILI episodes (*n* = 39), 54 % were accompanied by myalgia, 26 % by joint pain, 36 % by diarrhea and 21 % by vomiting. A doctor’s visit due to ILI was reported in 44 % (14/32) of cases. Diagnostic confirmation of the cause was not available. The incidence of first episode of ILI was 0.24 per 100 person-weeks of travel (95 % CI: 0.17-0.34) and of fever >38 °C, 1.6 per 100 person-weeks (95 % CI:1.4–1.8), similar in early weeks of travel (i.e. the first 12 weeks) and late travel periods. Any influenza virus infection was serologically confirmed in 38 of the 209 travelers with fever (PPV_fever_ = 18 %) and 12 of 32 travelers with ILI (PPV_ILI_ = 38 %).

Overall, 90 travelers seroconverted (attack rate = 15 %): *n* = 53 (9 %) seroconverted for 1 virus only (half were H1N1pdm09); 26 (4 %) for 2 viruses (16 were for both H3N2 viruses, two related strains where cross-reactivity was expected); 7 for 3 viruses and 4 people for 4 viruses. The only factor associated with any seroconversion was age (Table 1), and per-virus, only in those who seroconverted for H1N1pdm where more seroconversions occurred in those aged 25–28 years (Table 1). Influenza transmission zone visited was not a significant factor in seroconversion (Table 1). The AR for symptomatic infection (fever and ILI) was 6.3 % (38/602) and 2 % (12/602), respectively. Travelers that seroconverted, particularly to H1N1pdm09, were more likely to complain of ILI. (OR_ref:H1N1pdm09_ 6.2 [95 % CI:2.6–14.4], Table 2). Seroconversion to an increasing number of viruses was positively associated with ILI (IRR_unit increase in virus seroconversions_: 2.4, 95 % CI:1.5–4.0, *p* = 0.001). No seasonality (data not shown) or differences across influenza transmission zones (Fig. 1) was found with ILI onset or fever >38°.

**Table 2:** Association between individual virus seroconversion and influenza like illness (ILI) during the study period (2009 to 2012)

Virus	Odds ratio^a^	95 % CI	*p* value
A/California/007/09 H1N1pdm	6,2	2.6–14.4	0.000
A/Brisbane/59/07	1,2	0.2–9.3	0.866
A/Brisbane/10/07	3,6	1.1–11.0	0.028
A/Perth/16/09	1,8	0.5–6.2	0.354
B/Florida/4/06	1.0	[−−-]	[−−-]
B/Brisbane/60/08	1.0	[−−-]	[−−-]

^a^Odds ratio of association between seroconversion with individual virus and complaining of ILI. Univariable logistic regression by virus

## Discussion

In this young cohort of long-term travelers to (sub) tropical countries (2009 to 2012), the attack rate for confirmed influenza virus infection was 15 %. This is higher than the 1–7 % found in other, mainly short-term traveler studies [1, 12, 13] and closely mirrors seasonal rates of infection in the (unvaccinated) general population in temperate [14] and tropical [15] regions during the same period, reflecting the prolonged travel duration. For symptomatic infection, we found attack rates of 6.3 % (fever alone) and 2 % (ILI), respectively, which was higher than in two short-term studies that found attack rates of 0.9–1.3 % for confirmed infections with fever alone [1, 12] and 0.8 % for ILI [12]. The results of symptomatic infections however, should be interpreted with care. First, some studies use different definitions than ILI for symptomatic infections and do not report fever as the only symptom of confirmed infection [13]. Second, fever is a very common symptom in travelers [1, 12]. At the same time, confirmed infections without fever were found in 32 and 83 %, respectively [1, 12]. Because virological data from nasopharyngeal sampling was not available for any of the studies, we can not be sure whether the symptoms were caused by influenza or by other infections. In our study, both the proportion of travelers with fever (35 %) and the proportion of travelers with confirmed infections without fever (58 %) were high. Therefore we did not calculate IR’s for symptomatic influenza infections.

As shown elsewhere [15], travelers in their mid-20s were more susceptible to A(H1N1pdm09) infection than other viruses. The majority of respondents were immune to at least one virus pre-travel, and 1/5th departing before the pandemic was declared were positive for A(H1N1pdm09) as early as January 2009. Forty percent of travelers were infected with >1 virus while traveling and 10 people experienced a reinfection. Travelers infected with A(H1N1pdm09) during travel were more likely to be symptomatic with ILI. Overall, the positive predictive value of ILI was 38 %. We found no evidence of seasonality or destination-specific risk, and travel was not a risk factor for severe disease.

There were some limitations to our study. The pre-travel influenza vaccination status was not confirmed. However, in the Dutch healthcare system, it is very uncommon that healthy, young travelers are vaccinated. Another possible limitation is that travelers only measured their temperature if they ‘felt feverish’ rather than daily. This may have lead to an underestimate of the proportion of travelers with fever, and an overestimate of the number of asymptomatic infections.

Vaccination for travelers against influenza has been discussed [16]. In the USA, influenza vaccination is recommended for all US residents aged ≥6 months [17]. The USA guidelines for international travel (Yellow Book, 2016) say ‘any traveler who wants to reduce the risk for influenza infection should consider influenza vaccination ≥2 weeks before departure if they plan to travel to the tropics’. According to WHO, influenza vaccination should be part of the routine immunization program for international travelers belonging to a risk group, in particular during influenza seasons [18].

In the Netherlands, only people at risk for complications are adviced influenza vaccination. Because there is no evidence that travel is an additional risk factor for influenza, the Dutch guidelines for travelers health advice do not recommend influenza vaccination for travelers other than those at high risk of complications.

## Conclusions

We found that the attack rate of influenza in long-term travelers closely mirrors seasonal rates in the general population in temperate and tropical regions and travel was not a risk factor for severe disease. Influenza vaccination pre-travel is therefore most important for people at risk of medical complications due to influenza.

### Ethical approval and consent to participate

The study was approved by the Medical Ethics Committee of the Academic Medical Center.

Pre-travel, written informed consent was obtained form all participants.

### Consent for publication

Not applicable.

### Availability of data and materials

Data will be shared upon request. Identifying/confidential patient data however will not be shared.

## Abbreviations

AR: attack rate
HI: hemagglutination-inhibition
ILI: influenza like illness
PPV: positive predictive value
